# Different Brain Activation after Acupuncture at Combined Acupoints and Single Acupoint in Hypertension Patients: An Rs-fMRI Study Based on ReHo Analysis

**DOI:** 10.1155/2019/5262896

**Published:** 2019-01-03

**Authors:** Jiping Zhang, Xiaowen Cai, Yanjie Wang, Yu Zheng, Shanshan Qu, Zhinan Zhang, Zengyu Yao, Guanghong Chen, Chunzhi Tang, Yong Huang

**Affiliations:** ^1^School of Traditional Chinese Medicine, Southern Medical University, Guangzhou, Guangdong Province 510515, China; ^2^Weinan Vocational and Technical College, Weinan, Shaanxi Province 714026, China; ^3^School of Chinese Medicine, LKS Faculty of Medicine, HKU, Hong Kong; ^4^Clinical Medical College of Acupuncture, Moxibustion and Rehabilitation, Guangzhou University of Chinese Medicine, Guangzhou, Guangdong Province 510405, China

## Abstract

**Background:**

Acupuncture is proved to be effective on hypertension by numerous studies and resting-state functional magnetic resonance imaging (Rs-fMRI) is a widely used technique to study its mechanism. Along with lower blood pressure, patients with hypertension receiving acupuncture also presented improvement in function of cognition, emotion, language, sematic sensation, and so on. This study was a primary study to explore the acting path of acupuncture at combined acupoints in stimulated brain areas related to such functions.

**Methods:**

In this research, regional homogeneity (ReHo) was applied to analyze the Rs-fMRI image data of brain activities after acupuncture at LR3, KI3, and LR3+KI3 and to compare the differences of functional brain activities between stimulating combined acupoints and single acupoint under pathological conditions. A total of thirty hypertension patients underwent Rs-fMRI scanning before acupuncture treatment and then were randomly divided into three groups following random number table, the LR3 group (3 males and 7 females), the KI3 group (3 males and 7 females), and the LR3+ KI3 group (4 males and 6 females) for needling, respectively. When the 30-min treatment finished, they received a further Rs-fMRI scanning. The Rs-fMRI data before and after the acupuncture treatment were analyzed through ReHo.

**Results:**

Compared with preacupuncture, respectively, ReHo values increased in Brodmann areas (BAs) 3, 18, and 40 and decreased in BAs 7 and 31 in LR3+ KI3 group. However, ReHo values only decreased in BA7 of KI3 group while the results showed no significant difference of brain regions in LR3 group between pre- and postacupuncture. Compared with LR3 group, LR3+KI3 group exhibited decreased ReHo values in BAs 7, 9, and 31. Meanwhile, compared with KI3 group, LR3+KI3 group exhibited increased ReHo values in the BAs 2, 18, 30, and 40 and decreased ReHo values in BA13.

**Conclusion:**

Combined acupoints of LR3 and KI3 could act on wider brain areas than the sum of single acupoints, whose functions include emotional processing, cognition, somatic sensation, spatial orientation, language production, and vision.

## 1. Background

Acupuncture is an essential part of Traditional Chinese Medicine (TCM). Nowadays, the effect of acupuncture has been admitted worldwide, and this therapy has become one of the representative complementary and alternative medicines. In clinical treatment, most of the acupuncture prescriptions are made up of at least two acupoints. Previous researches have proven that the overall efficacy of acupoints combination is better than that of single acupoint [1, 2]. Researchers not only indicated that combination of acupoints had advantage over single acupoint from macroscopic perspectives [3], but also proved it at molecular level with animal experiments [4]. In the relative researches, the most widely used technique is functional magnetic resonance imaging (fMRI). Some scholars have confirmed with Rs-fMRI (resting-state functional magnetic resonance imaging), one method of fMRI, that the therapeutic effect of combination of acupoints is not a simple accumulation of several single acupoints but synergistic effect by influencing more brain regions than the sum of single acupoints, and the altered regions are different from the results of single acupoints groups too [5].

As a noninvasive, real-time, and visible new method, fMRI has been increasingly applied to explore the central mechanisms of acupuncture. Previously, many acupuncture researches based on Block Design fMRI came up with meaningful conclusions, including preliminarily confirming the existence of acupoint specificity and correlation among acupuncture, acupoints and brain [6, 7]. Nevertheless, these experiments with Block Design fMRI cannot represent actual clinical acupuncture treatment process, compared with Rs-fMRI, a technology that can simulate physiological status of a person better and reflect complicated functional activities of brains truly.

In Rs-fMRI studies, regional homogeneity (ReHo) is one of the frequently used methods to analyze image data of brain activities. In ReHo analysis, Kendall's coefficient concordance (KCC) is used to measure the similarity of a number of time series of a given voxel to those of its nearest neighbors in a voxel-wise way. By assessing the local synchronization of the Rs-fMRI signals, the method of ReHo can reflect the synchronous activities of a specific brain region [8]. It helps to promote the understanding of the complexity of brain function.

To date, a growing number of Rs-fMRI studies have thrown light on the mechanisms of acupoints combination under healthy state. Based on fMRI, Liu et al. found that ST36 (Zusanli) and GB34 (Yanglingquan) together could activate more brain areas and generate new curative effects compared to single acupoint [9]. Chen et al. and Cai et al. suggested that the change of cerebral regions caused by acupoints combination may be connected with the cooperative integration effect [1, 10]. However, most of the experiments about acupoints combination were applied to healthy subjects. It has been proven that brain functional activities were different in normal people and patients through Rs-fMRI scan [11]. Actually, the mechanism of acupoints combination under pathological state is different from healthy state. Therefore, based on our former researches, we chose patients as subjects in this experiment to seek the evidence.

So far, current Rs-fMRI researches studying the mechanism of acupoints combination on diseases can be basically divided into two kinds. Ones are based on conventional therapy. For example, Li et al. observed increased functional connectivity of acupuncture in addition to western medicine group from pre- and posttreatment for stroke [12]. The others compared either the differences of functional brain activities changes between acupoints combination and sham acupoints on patients [13] or acupoints combination manipulation on patients and the healthy controls [14, 15]. Reviewing the recent studies, there is a substantial lack of comparison between acupoints combination and single acupoint in brain activities observation. The animal trial of Wang et al. is one of the few that focused on this aspect, which found that the principal-subordinate acupoints combination of PC6 (Neiguan), ST36, and CV4 (Guanyuan) could enhance the expression of the PI3K/Akt signaling pathway compared to PC6 group, which was beneficial to the myocardial tissues [4].

LR3 (Taichong) and KI3 (Taixi) are one of the most commonly adopted paired acupoints in clinical treatment such as infantile enuresis [16], decline in ovarian reserve [17], hand spasm in stroke [18], etc. And it has been proven in the animal trail of Xia et al. that the combination of LR3 and KI3 could upregulate the expression neuroprotective factors in hippocampus of rats after cerebral ischemia injury and improve their learning and memory abilities [19]. During the literature searching and clinical observation, we found that both LR3 and KI3 are also commonly used in clinical acupuncture treatment for hypertension, and meta-analysis has provided evidence that acupuncture has therapeutic effect on hypertension as an adjunctive therapy to medication [20, 21], too.

Rs-fMRI is gradually used in studying effect of acupuncture on human brains with hypertension. Zhu et al., discovered that acupuncture at KI3 had a specific effect on some brain regions which were in charge of perception, visual and auditory sense, body movement, spirit, association, etc. [22]. Sun et al., considered that LR3 could activate anterior cingulated gyrus to regulate parasympathetic nerve and lower BP (blood pressure) [23], and Zheng et al., found that LR3 could induce brain functional connectivity in the frontal lobe, cerebellum, and insula, which made up of a possible neural network structure with specific functions of acupuncture in treating hypertension [24].

Although some researches have studied the mechanism of LR3 or KI3 on hypertension, these two acupoints were utilized separately [20, 23, 24]. Our earlier Rs-fMRI researches carried out experiments of acupuncture combination of LR3 and KI3 [25, 26], single LR3 [27], and single KI3 [22], but most of the subjects were healthy humans. One of our studies corroborated the synergistic effect of acupoints combination in lowing blood pressure and stimulating more brain regions. To further analyze the differences of functional brain activities aroused by acupoints combination and single acupoints and possible acting path of acupuncture at combined acupoints in the changed brain areas under pathological conditions, patients with hypertension were recruited in this experiment and were divided into three groups undergoing different acupuncture therapy, with acupoints combination of LR3 and KI3 and with single LR3 or KI3.

## 2. Method

### 2.1. Subjects

Thirty hypertension patients (10 males and 20 females), aged 40–65 years, were included in this study. Subjects weighed 46–72 (55.40 ± 8.35) kg and were 160–180 (168.6 ± 6.81) cm tall. All subjects gave full informed consent before the experiment. This study was approved by the Chinese Ethics Committee of Registering Clinical Trials (ChiECRCT-2012011) and registered in the Chinese Clinical Trial Register (ChiCTR-TRC-12002427).

### 2.2. Inclusion Criteria

Inclusion criteria are as follows: (1) Patients with grade 1 or 2 hypertension, taking antihypertensive medicine regularly and maintaining a stable BP during the experiment. (2) Aged 40–65 years, right-handed. Male or female is not limited. (3) Compliant with the diagnostic criteria and risk stratification standard in hypertension guidelines revised and published by World Health Organization (WHO)/ International Society of Hypertension (ISH) [28]. (4) Having not received acupuncture within 1 month before the test. (5) All the subjects signed the appropriate informed consent paperwork and were willing to cooperate in the experiment.

### 2.3. Exclusion Criteria

Exclusion criteria are as follows: (1) Having been diagnosed with cerebrovascular accident and/or combined with other serious diseases, or acupuncture contraindications such as coagulation disorders, haemophilia, etc. (2) Females in pregnancy or lactation and patients with mental diseases. (3) Acupoint site with skin damage or disease. (4) Having suffered from different pain including dysmenorrhea within 1 month, which may affect therapeutic effect. (5) Having fainted before during acupuncture. (6) Having received acupuncture within 1 month before the test. (7) Having intense reaction to noise or in low-temperature environment and claustrophobic.

### 2.4. Procedure Outline

**Figure 1:**
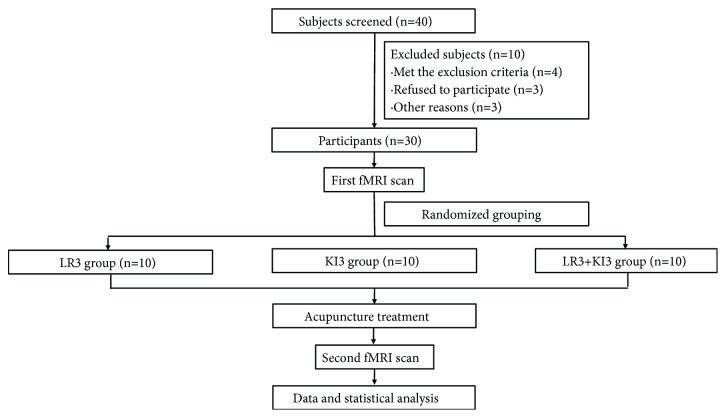
Flowchart of the participants. fMRI: functional magnetic resonance imaging.

**Figure 2:**
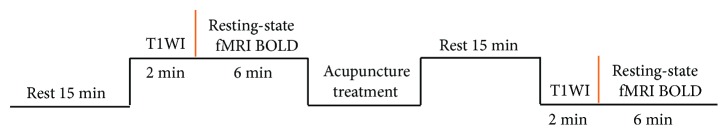
Flowchart of the experimental procedure. T1WI: Transverse T1-weighted image; BOLD: blood-oxygen-level dependent data; min: minutes.

Subjects were asked to pass urine and stool prior to experiment. After resting for 10 min, each of them accepted an Rs-fMRI scan before the acupuncture treatment. All of the subjects were then randomly divided into three groups following random number table--the LR3 group (3 males and 7 females), the KI3 group (3 males and 7 females) and the LR3+ KI3 group (4 males and 6 females), and received needling lasting for 30 minutes. 15 minutes after the needles withdrawn, a further Rs-fMRI scan was performed. The Rs-fMRI data before and after the acupuncture treatment were analyzed through ReHo. The flowcharts of participants and the experimental procedure are shown in Figures 1 and 2.

### 2.5. LR3 and KI3 Location

**Figure 3:**
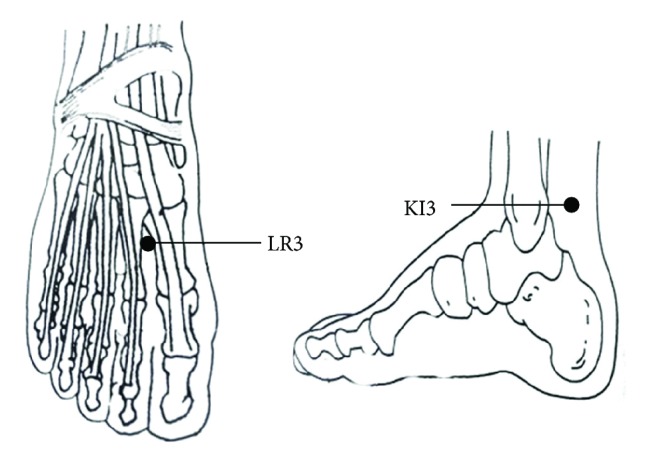
The location of LR3 and KI3.

LR3 is located in the depression anterior to the junction of first and second metatarsal bones and KI3 is located in the depression between the tip of the medial malleolus and the Achilles tendon according to Name and Location of Acupoints: Chinese National Standards (GB/T 12346-2006) (Figure 3). In this experiment we used acupoints at both of the sides of the participants.

### 2.6. Acupuncture Treatment

All acupuncture manipulation was performed by the same qualified and experienced doctor. The whole treatment continued for 30 min. Needling was performed as follows: after the overlying skin was sterilized, sterile acupuncture needles (25.0 mm × 0.3 mm; HwaTo; Suzhou Medical Supplies Co., Suzhou, Jiangsu Province, China) were inserted perpendicularly into the bilateral acupoints of all subjects to a depth of about 12.5 mm with uniformly twisting, lifting and thrusting. In the LR3+KI3 group, needles were firstly inserted into bilateral LR3. Once the patients got acupuncture sensation (feelings like soreness, numbness, heaviness, fullness, etc.), needling was manipulated at KI3 under the same procedure. All needling was performed from the participants' left to the right sides. The needle was manipulated for 1 min at intervals of 10 min during the treatment.

### 2.7. Resting-State fMRI Scanning

Resting-state fMRI (Rs-fMRI) data were collected by a GE 3.0T MRI scanner (Signa Excite System, General Electric Medical Systems, Milwaukee, WI, USA) with an 8-channel head coil. During the scanning, the subjects' sense of vision and hearing was blocked by wearing blindfolds (Hanjiang Xinhua Tourist Supplies Factory, Yangzhou, Jiangsu Province, China) and earplugs (Aearo, Indianapolis, IN, USA) to avoid audio-visual interference. They were also told to be relaxed and breathe peacefully with their heads fixed by foam headrests to reduce active or passive movements. The scanning procedure was performed as follows: 

*(1) Transverse T1-Weighted Image (T1WI) Sequence*. 2 minutes, fast spin echo sequence; OAx T1 FLAIR, repetition time = 1,750 ms, echo time = 24 ms, inversion time = 960 ms, field of view = 24 × 24 cm2, matrix = 320 × 224, number of excitations = 1, thickness = 5.0 mm; interval = 1.0 mm; slice layers = 30; echo train length = 8; bandwidth = 31.25. 

*(2) Resting-State fMRI Blood-Oxygen-Level Dependent Data Collection*. Gradient echo-echo-planar imaging sequence scanning was used for 6 minutes, with the following scan parameters: repetition time = 3,000 ms/minimum, echo time = minimum; flip angle = 90°; field of view = 240 mm × 240 mm; thickness = 5.0 mm; interval = 1.0 mm; slice layer = 30 slices per acquisition; matrix = 96 × 96; number of excitations = 1.

### 2.8. Image Processing

Image preprocessing was conducted by Data Processing Assistant for resting-state fMRI (DPARSF) (Yan and Zang, 2010, http://www.restfmri.net) based on Statistical Parametric Mapping (SPM8, http://www.fil.ion.ucl.ac.uk/spm) and the resting-state fMRI Data Analysis Toolkit (REST, Song et al., 2011, http://www.restfmri.net) [29, 30] on Matlab R2009a platform. This process contained 6 steps: DICOM format conversion, removal of 10 time points prior to image scanning, time correction, correction of head movement, spatial standardization and spatial smoothing. The data of subjects that three-dimensionally panned > 1.5 mm and (or) rotated > 1.5° were removed. Spatial standardization was finished with the MNI template developed by the Montreal Neurological Institute, Canada. After finishing the fitting between the MNI template and the 3 mm x 3 mm x 3 mm sample, the data with poor standardized fitting were excluded by observing the degree of standardization.

### 2.9. Data and Statistical Analysis

Data and Statistical analysis was accomplished by REST1.8 software [30] (http://www.restfmri.net/forum/REST_V1.8). 

*(1) ReHo Analysis*. After spatial standardization, the linear tendency of the preprocessed data was removed by linear regression method. Then nuisance covariates regression was performed, and covariates included head motion with ripid-body 6 head model, white matter signal, and cerebrospinal fluid signal. After that, the Kendall's coefficient of concordance (KCC) value of each voxel was calculated to obtain an individual KCC map or ReHo map. These maps underwent whole-brain equalization for further statistical analysis [8, 29, 31]. 

*(2) Statistical Analysis*. Intragroup standardized values of the ReHo were evaluated by a paired* t*-test (*α* = 0.05, AlphaSim correction* P *< 0.05, continuous voxel > 85). Once the differences of the ReHo between preacupuncture and postacupuncture in each group were acquired, the specific anatomical position of brain areas corresponding to the MNI coordinate could be identified using Rest1.8 software Viewer, and the results were presented by images. The paired* t*-test was used for intragroup comparison before and after acupuncture, respectively, and one-way analysis of variance (ANOVA) or Kruskal-Wallis H was used for comparison among three groups with SPSS 20.0 (SPSS Inc., Chicago, IL, USA).

## 3. Results

### 3.1. Changes of Brain Regions in Each Group after Acupuncture

**Figure 4:**
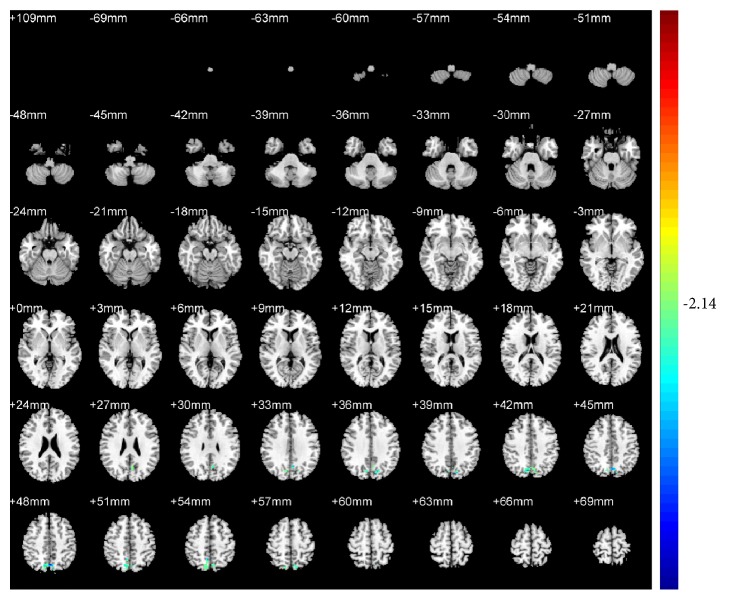
Changes of brain regions in KI3 group of posttreatment versus pretreatment.

**Figure 5:**
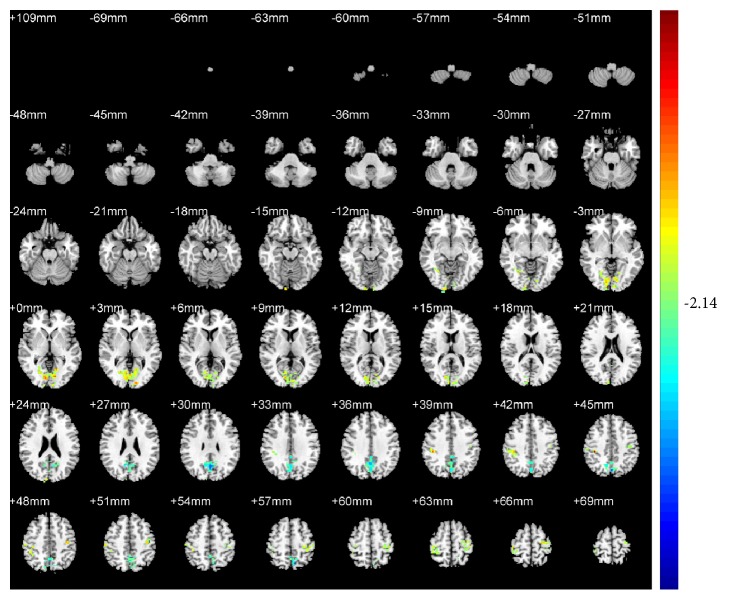
Changes of brain regions in LR3+KI3 group of posttreatment versus pretreatment.

Compared with pretreatment, KI3 group exhibited decreased ReHo (T value was negative) in the precuneus of the left parietal lobe (BA7) (Table 1 and Figure 4). LR3+KI3 group exhibited increased ReHo (T value was positive) in the lingual gyrus of the left occipital lobe (BAs 18 and 3) and the postcentral gyrus of the parietal lobe (BA40), and decreased ReHo in the precuneus of left parietal lobe (BAs 7 and 31) (Table 2 and Figure 5). However, the results showed that there was no significant difference of brain regions in LR3 group between pre- and postacupuncture.

### 3.2. Comparison of Changes of Brain Regions among Three Groups after Acupuncture

**Figure 6:**
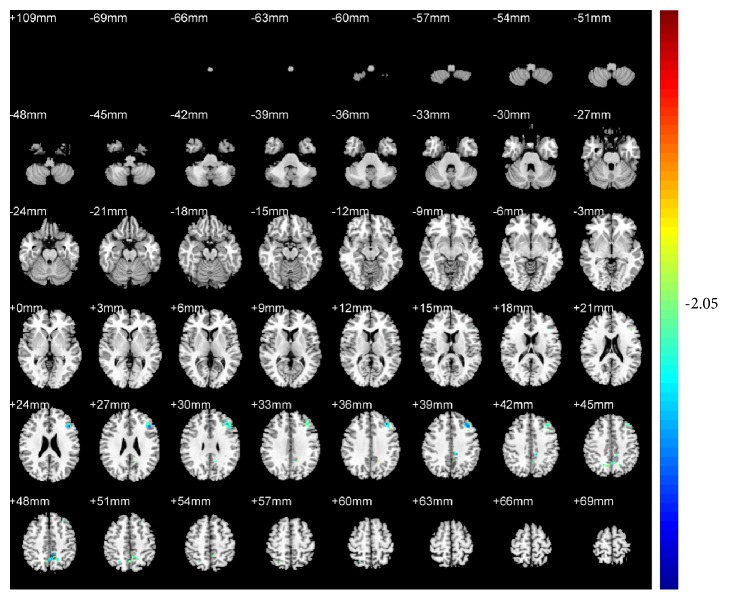
Differences of brain regions between LR3+KI3 and LR3 group of posttreatment versus pretreatment.

**Figure 7:**
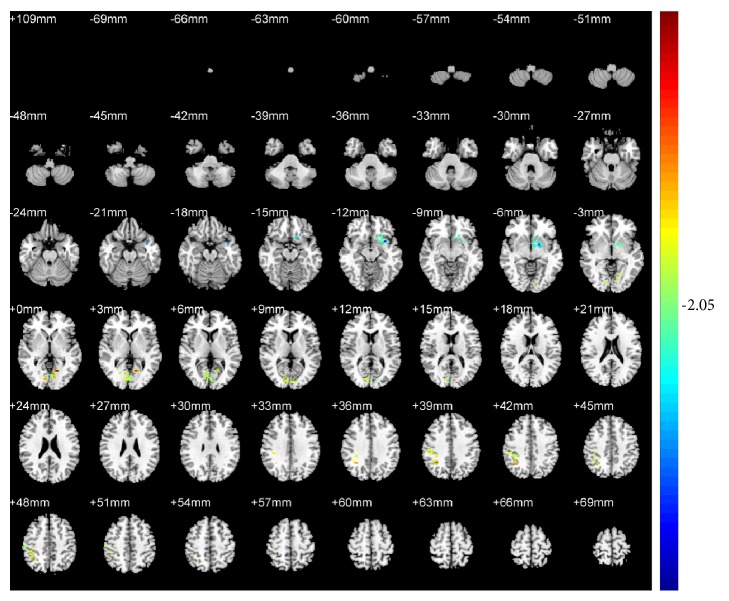
Differences of brain regions between LR3+KI3 and KI3 group of posttreatment versus pretreatment.

Compared with LR3 group, LR3+KI3 group exhibited decreased ReHo in the left superior frontal gyrus (BA9) and the precuneus of the left parietal lobe (BAs 7 and 31) (Table 3 and Figure 6). Meanwhile, compared with KI3 group, LR3+KI3 group exhibited increased ReHo in the lingual gyrus of the left occipital lobe (BAs 18 and 30) and the right inferior parietal lobule (BAs 40 and 2), and decreased ReHo in the left inferior frontal gyrus (BA13) (Table 4 and Figure 7).

## 4. Discussion

Since the efficacy of LR3 and KI3 has been verified in clinical practice [4, 16, 26, 32, 33], some Rs-fMRI researches have been carried out to investigate the mechanism underlying these paired acupoints on treating different diseases. For example, Tan et al., observed that acupuncture at LR3 and KI3 with other acupoints activated connectivity between cognition-related regions including insula, dorsolateral prefrontal cortex, hippocampus, thalamus, inferior parietal lobule, and anterior cingulate cortex through Rs-fMRI scanning [32]. Yet the experiments applied to patients maintain inadequate.

One of our former researches testified the curative effect of combination of LR3 and KI3 on hypertensive patients with positive results-reduced BP and improved symptoms after acupuncture treatment-in the experiment of Wang et al. [5]. On the mechanism of LR3 and KI3 concentrating on healthy controls, we discovered that the efficacy of this acupoints combination was connected with certain specific brain regions like the frontal lobe, the cerebellum, and the occipital lobe, which were more than the totality of the brain regions stimulated by single acupoints [25, 34]. In this study, we further investigated the action mechanism of LR3 and KI3 on patients with hypertension.

More and more studies have tried and contributed to revealing the potential mechanism of acupuncture on antihypertensive effect and symptom improvement. Systematic analysis of changes of functional brain regions under pathologic condition and after acupuncture has been one of the latest trends in hypertension researches. At present, it has been corroborated that acupuncture can activate several functional brain networks such as default mode network, pain modulating system, limbic system, etc. [35–37]. As for hypertension, brain regions stimulated by acupuncture are mainly related to functions of cognition, emotion and mood, memory, language, sensory and motor processing and so on [5, 23, 25]. In spite of high blood pressure, many patients with hypertension suffer from dizziness, headache, numbness of limbs, palpitations, and cognitive dysfunction at the same time. Acupuncture can not only lower blood pressure, but also improve other concomitant symptoms [38]. The mechanism may be connected with the alternated brain regions mentioned above.

The results of this experiment reflected that the effect of combined acupoints was not a simple sum of two single acupoints' effect. Rather, the combined acupoints created a broader stimulation of brain areas. After acupuncture treatment, the LR3+KI3 group demonstrated more altered functional brain regions than LR3 group or KI3 group with three activated brain regions--BAs 3, 18 and 40, and two deactivated brain regions--BAs 7 and 31, among which the activation constituted the majority. These brain areas are related to the function of emotional processing and recognition, cognition, somatic sensation, spatial orientation, semantic representation and language production, vision, etc. Meanwhile, acupuncture at KI3 only deactivated one brain area and acupuncture at LR3 recorded no significant results compared with pretreatment. This phenomenon also showed up in experiment of Wang et al., and was considered as a synergistic effect of combined acupoints [5].

After analysis, ReHo values of BAs 9 and 31 decreased in LR3+KI3 group versus LR3 group and that of BA13 decreased in LR3+KI3 group versus KI3 group. The deactivation of BAs 9 and 13 was also observed in the study of Liu et al. [9] concentrating on the effect of acupoints combination comprising ST36 and GB40 (Yanglingquan) on healthy controls. BAs 9, 13 and 31, partitions of the frontal lobe and the upper posterior cingulate cortex, are mainly connected with cognitive function as well as emotional processing and recognition. In the study of László et al. affective temperaments were associated with the pathogenesis of hypertension, suggesting that patients who presented high cyclothymic temperament were in a higher risk of elevated blood pressure [39]. What is more, before evolving into stroke, there has been an impairment of the brain morphology and cognitive function of patients with hypertension to a certain degree, leading to depression or anxiety in future disease progression [40, 41]. Findings above reflected the interrelationship between hypertension and cognitive and emotional disorders. Therefore, the alteration of BAs 9, 13, and 31 may be targeting brain areas of acupoints combination controlling blood pressure through cognition and emotion regulation.

In contrast, acupuncture at LR3+KI3 activated BAs 3 and 40 compared with pretreatment and BA2 compared with KI3 group. Located in somatosensory cortex, BAs 2 and 3 are in charge of somatic sensory sensations. BA40, situated in supramarginal gyrus, is a part of Wernicke's area and belongs to secondary somatosensory cortex. In addition to its chief function of semantic representation and language production, it also plays a role in spatial orientation. Considering the symptoms like numbness of limbs and dizziness in some hypertensive patients, the activation of these three brain regions may be assistant to the improvement of therapeutic effect induced by acupoints combination as a benign compensatory regulation.

Besides, we also observed alteration in BAs 7, 18 and 30 after acupuncture in interior-group and intergroup comparison. Since BA7 was deactivated in both single acupoint group (KI3 group) and combined acupoints group compared with pretreatment, respectively, we deem that it may be remote from the specificity of acupoints combination. Both BAs 18 and 30 are located in the occipital lobe, whose primary structure is visual cortex center. Some researches indicated that visual cortex was associated with emotional activity and modulation [42, 43]. In view of the insufficient researches discussing the relationship between the occipital lobe and the improvement of clinical symptoms on patients with hypertension, we suppose that acupuncture at combined acupoints altered more brain regions.

The research still had some limitations. Firstly, without healthy control group, the changes of brain activities were compared with the baseline. Secondly, it lacked the correlation analysis between altered blood pressure and changed ReHo value regions. Thirdly, the needling manipulation was applied only once to patients of each group in our experiment. Consequently, the diversity of acupuncture effect on each patient may lead to different strength and proportion of changed brain regions. Besides, due to the different conditions of patients, the antihypertensive medicine therapy of each subject uncontrollable issues, including course of treatment, kinds and dosage of drugs, etc. In the future, we need to carry on long-term acupuncture treatment on hypertensive patients and enlarge the sample size for a profounder understanding of the mechanism of acupoints combination under pathologic state.

## 5. Conclusion

To sum up, we used acupoints combination in treating patients with hypertension and applied ReHo method to analyzing the Rs-fMRI data to compare the functional brain areas stimulated by combined acupoints and single acupoints. Combined acupoints of LR3 and KI3 could act on wider brain areas than the sum of single acupoints, whose functions include emotional processing, cognition, somatic sensation, spatial orientation, language production, and vision.

## Figures and Tables

**Table 1:** Changes of brain regions in KI3 group of posttreatment versus pretreatment.

Cluster	Voxels	Brain regions	Side	Brodmann area	T value (peak intensity)	Peak MNI coordinate (mm)		
						X	Y	Z
1	141	Parietal lobe, precuneus	Left	7	-5.251	-3	-69	48

**Table 2:** Changes of brain regions in LR3+KI3 group of posttreatment versus pretreatment.

Cluster	Voxels	Brain regions	Side	Brodmann area	T value (peak intensity)	Peak MNI coordinate (mm)		
						X	Y	Z
1	422	Occipital lobe, lingual gyrus	Left	18	6.5749	-12	-78	0
2	316	Left parietal lobe, precuneus	Left	7, 31	-5.5874	0	-72	45
3	123	Parietal lobe, postcentral gyrus	Left	3	5.1571	-42	21	51
4	130	Right parietal lobe, postcentral gyrus	Right	40	7.2998	39	-30	45

**Table 3:** Differences of brain regions between LR3+KI3 and LR3 group of posttreatment versus pretreatment.

Cluster	Voxels	Brain regions	Side	Brodmann area	T value (peak intensity)	Peak MNI coordinate (mm)		
						X	Y	Z
1	137	Left superior frontal gyrus	Left	9	-4.3329	-36	33	36
2	86	Left parietal lobe, precuneus	Left	7, 31	-3.5879	-6	-45	48

**Table 4:** Differences of brain regions between LR3+KI3 and KI3 group of posttreatment versus pretreatment.

Cluster	Voxels	Brain regions	Side	Brodmann area	T value (peak intensity)	Peak MNI coordinate (mm)		
						X	Y	Z
1	150	Left occipital lobe, lingual gyrus	Left	18, 30	4.7685	-15	-63	0
2	92	Left inferior frontal gyrus	Left	13	-3.941	-33	12	-12
3	120	Right inferior parietal lobule	Right	40, 2	4.1843	36	-51	39

## Data Availability

The original data used to support the findings of this study are included within the article.
